# Partnered Dancing to Improve Mobility for People With Parkinson's Disease

**DOI:** 10.3389/fnins.2015.00444

**Published:** 2015-12-11

**Authors:** Miek J. de Dreu, Gert Kwakkel, Erwin E. H. van Wegen

**Affiliations:** ^1^Department of Neurosurgery, Clinical Imaging Tilburg, Elisabeth HospitalTilburg, Netherlands; ^2^Department of Rehabilitation Medicine, MOVE Research Institute Amsterdam, VU University Medical CenterAmsterdam, Netherlands

**Keywords:** Parkinson's Disease, dance, music, recreational therapy, Salsa, Tango, exercise

## Introduction

Parkinson Disease (PD) is a neurodegenerative disease for which no cure is available yet. It is the second largest neurological disease affecting an estimated 571 per 100,000 people in Europe with rising prevalence due to the aging population (Pringsheim et al., 2014). To date, dopamine-replacement therapy (DRT) is the first choice of treatment to lessen the impact of motor and non-motor symptoms, however DRT does not prevent progressive disabilities and does not change the course of the disease (Chaudhuri et al., 2006; Jankovic and Stacy, 2007). Therefore, other types of therapy are needed to supplement DRT.

Recent evidence suggests that physical activity and vigorous exercise may have the potential to slow disease progression (for an overview see Hirsch and Farley, 2009; Ahlskog, 2011; van Wegen et al., 2014). These findings are promising, however they require further investigation. The effects of physical activity and regular exercise on reducing the chance of developing secondary problems e.g., diabetes or cardiovascular disease are quite established (Lee et al., 2012). Nevertheless, inactivity is a major problem as patients with PD are approximately one third less active than age matched controls (van Nimwegen et al., 2011). Being physically active may be more difficult for patients with PD because of physical impairments, fatigue, and apathy (van Nimwegen et al., 2011). Physical rehabilitation, containing a variety of exercise interventions (e.g., individual and in groups) is recommended for patients with PD (Keus et al., 2014).

### Partnered dancing

The European guideline for Parkinson's disease recommends dance as a meaningful approach to improve functional mobility and balance (Keus et al., 2014). However, this recommendation is based only on three proof-of-concept trials that investigate Tango dancing (Hackney et al., 2007; Hackney and Earhart, 2009a; Duncan and Earhart, 2012).

Several reviews have been published that included more studies regarding music based movement therapy and dance (de Dreu et al., 2012, 2014; Sharp and Hewitt, 2014; Shanahan et al., 2015a). A recent meta-analysis including five randomized clinical trials suggests significant positive effects of dance therapy on motor impairment, balance, gait speed, and health-related quality of life (Sharp and Hewitt, 2014). A systematic review investigating multiple types of dance found significant positive effects on endurance, motor impairment, and balance (Shanahan et al., 2015a). Furthermore, our meta-analysis investigating several types of music based movement therapies (dance and gait-based interventions using music as an auditory rhythmic cue) found significant positive effects on balance performance, UPDRS-II, walking velocity, stride length, dual task walking velocity, 6 m walk test, and the timed-up-and go test (de Dreu et al., 2012, 2014).

Most studies on dance in patients with PD have investigated Tango dancing (Hackney et al., 2007; Hackney and Earhart, 2009a,b,c, 2010a,b; Duncan and Earhart, 2012; Foster et al., 2013; McKee and Hackney, 2013; Duncan and Earhart, 2014). In addition, one study on Ballroom dancing (Hackney and Earhart, 2009a,b) and two pilot studies on Irish set dancing (Volpe et al., 2013; Shanahan et al., 2015b) were published. Salsa dance classes for patients with PD are available in the Netherlands and in Canada (Ottawa). Furthermore, there are organizations in several countries providing modified modern dance classes and/or ballet classes (not partnered dancing) for patients with PD, e.g., Dance for PD (New York), Dance for Parkinson's (London), Queensland ballet, and Dance for Health (multiple cities in The Netherlands). Some small pilot studies have investigated these types of dance (Westbrook and McKibben, 1989; Batson, 2010; Heiberger et al., 2011), however the effectiveness of Salsa dance classes remains to be established in methodologically well-conducted randomized controlled trials.

Partnered dancing combines exercise with cognitive challenges in an enriched environment with (somato) sensory cues from the music as well as from the dance partner (Bläsing et al., 2012). The sensory cues from physical contact with the partner are specifically important during Tango and Salsa dancing. While Ballroom and Irish set dancing often have a predefined routine that is executed from start to end in the way that people are required to learn the entire routine by heart, Tango and Salsa dancing do not necessarily have such a routine, providing more flexibility in performance. During Tango and Salsa classes, participants are taught several short steps with specific somatosensory cues (signals) for each step (e.g., with a length of 8 or 16 counts in the music). Subsequently, the couple can apply these steps in any sequence. A dancing couple consists of a leader (traditionally the man) and a follower (traditionally the woman). However, this format is sometimes changed, e.g., in the studies about Tango dancing men and women practiced both the leading and following roles (Hackney and Earhart, 2010b). The leader determines which routine comes next and the follower responds to the somatosensory cues of the leader. This requires clear communication. An example in this context is a right turn for the follower, this can be indicated by the leader by raising the hand of the follower gently above his/her head, indicating the direction of the turn by choosing a spot just right or left from the center of his/her head. We advise the follower to turn with small steps in their own tempo and the leaders to follow the tempo of the followers. Some of these steps in Salsa and Tango are similar to physiotherapeutic strategies and training for weight shifting, turning, and backwards walking (Kamsma et al., 1995; Earhart, 2009). Consequently, there is a relatively high demand of planning skills for the leader and the responsiveness to somatosensory cues for the follower. In line with these observations, McKee and Hackney found that spatial cognition and executive function improved after 10 weeks of Tango dancing classes (McKee and Hackney, 2013). This finding is important in light of the decline of spatial cognition in neurodegenerative disease (Possin, 2010). These interactions resemble those with caregiver-mediated exercises after stroke (Galvin et al., 2011; Vloothuis et al., 2014) and may improve not only the functional mobility of patients but also decrease feelings of caregiver burden through mechanisms of empowerment and self-management. However, the effect of partnered dance on caregiver burden needs further investigation.

## Balance

Balance instability is one of the cardinal signs of PD (Kim et al., 2013) that responds poorly to and may even be worsened by DRT (Konczak et al., 2009). Balance problems and the related fall risk affect the daily life of patients with PD to a large extent and may prevent patients from being active (Wielinski et al., 2005; Abendroth et al., 2012).

Partnered dancing interventions have consistently improved balance performance without focusing on balance deficits (Earhart, 2009; de Dreu et al., 2012, 2014; Sharp and Hewitt, 2014; Shanahan et al., 2015b). The use of multiple types of sensory information simultaneously (e.g., auditory, somatosensory and proprioception) has been indicated as a critical aspect of balance control for patients with PD (Konczak et al., 2009; Conradsson et al., 2012; Lefaivre and Almeida, 2015). The predefined steps with stops, starts, changes in direction, and backwards stepping may provide practice of motor agility, which is another critical aspect of balance control and gait initiation/termination affected in patients with PD (Conradsson et al., 2012). An improvement of balance performance may enhance activities of daily living (ADL; Tan et al., 2012), health-related quality of life (Ellis et al., 2011), and with that, well-being of patients with PD.

## Music in partnered dance

Music is an integral and essential part of partnered dancing that provides a rhythm as well as an emotional context via a complex structure (e.g., loudness, pitch, timbre, harmony, melody, duration of the tone etc.; Krumhansl, 2000).

Usually, the type of music is specific for the type of dance. The rhythm of the music provides a timeframe, aiding in movement execution similarly as auditory cueing, provided that the patient with PD recognizes the rhythm (Keus et al., 2007; Nieuwboer et al., 2007; de Bruin et al., 2010; de Dreu et al., 2014; van Wegen et al., 2014). An important aspect of music in this context is the “groove.” The groove has been defined as the property of music that compels the body to move (Janata et al., 2012). Salsa music to date has not been investigated for groove, however Samba music (also Latin music) was found to contain a high level of groove (Madison et al., 2011). The structure of music (especially high groove and familiar music) may aid in synchronization with the rhythm compared to the isochronous beat of a metronome (Thaut et al., 1997; Janata et al., 2012; Getz et al., 2014; Leow et al., 2014; Hove and Keller, 2015). This is consistent with general functional perspectives of rhythmic music enabling and facilitating entrainment and precise synchronization of movements (Madison et al., 2011) and may be specifically important for patients with PD because of the problems in sensory-motor timing (Lucas et al., 2013; Hove and Keller, 2015).

Patients with PD may have some more difficulty in detecting the beat, acknowledging that the beat-based rhythm perception is worse when compared to controls (Grahn, 2009). Initially the majority of people (either Parkinsonian or healthy) may find it difficult to synchronize their steps to the rhythm of music, which may be related to a low familiarity with the music (Leow et al., 2014) and the cognitive load of timing the newly learned dance routines to the music (McKee and Hackney, 2013). Another explanation is that motor learning is required for proper timing of movements to external stimuli. Impairments in timing may decrease with improved sequential movement performance as a consequence. Further research on this aspect is needed.

Finally, music provides an emotional context and may temporarily alter mood (Krumhansl, 2000; Laukka, 2006; Zentner et al., 2008) through activation of specific brain areas such as amygdala, nucleus accumbens, hypothalamus, hippocampus, insula, cingulate cortex, and orbitofrontal cortex (Blood and Zatorre, 2001; Koelsch, 2014). This activation includes the release of several biochemical mediators (e.g., endorphins, endocannabinoids, dopamine, and nitric oxide; Boso et al., 2006). These neurophysiological aspects of music may increase therapy compliance for long-term interventions and distract from sensations such as fatigue during exercise (Hayakawa et al., 2000; Lim et al., 2011; Stork et al., 2015).

## Safety

Directly related to implementing a challenging dance training in a community setting is the risk for falling during the intervention. Partnered dancing is potentially a safe intervention. Provided the partner is strong enough, he or she may be able to provide physical support when necessary. Several other measures that may prevent falling in a dance class have been described by Hackney and Earhart (2010b). Safety largely depends on the skill of the dance-teachers to adequately adjust the difficulty of the steps to the ability of the participants. A recent small feasibility study of Tango dancing (*N* = 6; 4 weeks of weekly dance classes) reported no adverse events during dance classes (Blandy et al., 2015). Feasibility studies of Irish set dancing (*N* = 22; 8 months of weekly dance classes) reported one single fall with no injury (Volpe et al., 2013; Shanahan et al., 2015b). Therefore, partnered dance can be regarded safe and feasible when following the guidelines of Tango dancing (Hackney and Earhart, 2010b). However, the backward steps during Tango may pose a larger risk for falls than other dances. The European PD-guidelines for physiotherapy suggests that backward stepping during Tango dancing may increase the risk of falling during the dance intervention and highlights the importance of an adequate selection of patients for this type of intervention (Keus et al., 2014).

## Social aspects of partnered dance

Partnered dancing is an exercise regimen that requires substantial interaction between the dance partners and incorporates group dynamics as participants may switch dance partners during the class. Activities peripheral to the dance class such as drinks during breaks and peer-interactions before and after class provide additional possibilities for social interaction which may improve adherence (Rosa et al., 2015). This aspect of dancing may be specifically important when aiming for preventing social isolation.

To conclude, partnered dancing in a community setting seems a viable way to exercise. It is an attractive form of exercise therapy for patients with PD because it naturally combines evidence based aspects of music, cueing techniques, motor learning, balance exercises, and physical activity while focusing on interaction and enjoyment between partners and the group. Although theoretically several arguments exist regarding the mechanisms of action and putative effects of partnered dance, future research could compare different dance styles with regard to safety and effectiveness in order to further the field of music based exercise therapy. Furthermore, the effect of partnered dancing on the wellbeing of the partner needs further research.

## Author contributions

MD wrote the manuscript. GK and EV provided feedback and suggestions throughout the writing process.

## Funding

GK has received research-funding from ZonmW (89000001/630000012), ParkinsonFonds (20094), and EU (ERC-291339). EV received research-funding from ZonMw (630000012), Parkinson Vereniging (2013R16), ParkinsonFonds (20094), and the Hersenstichting (F2013141).

### Conflict of interest statement

The authors declare that the research was conducted in the absence of any commercial or financial relationships that could be construed as a potential conflict of interest.
